# Variables to Be Monitored via Biomedical Sensors for Complete Type 1 Diabetes Mellitus Management: An Extension of the “On-Board” Concept

**DOI:** 10.1155/2018/4826984

**Published:** 2018-09-30

**Authors:** Ignacio Rodríguez-Rodríguez, José-Víctor Rodríguez, Miguel-Ángel Zamora-Izquierdo

**Affiliations:** ^1^Universidad de Murcia, Spain; ^2^Universidad Politécnica de Cartagena, Spain

## Abstract

Type 1 diabetes mellitus (DM1) is a growing disease, and a deep understanding of the patient is required to prescribe the most appropriate treatment, adjusted to the patient's habits and characteristics. Before now, knowledge regarding each patient has been incomplete, discontinuous, and partial. However, the recent development of continuous glucose monitoring (CGM) and new biomedical sensors/gadgets, based on automatic continuous monitoring, offers a new perspective on DM1 management, since these innovative devices allow the collection of 24-hour biomedical data in addition to blood glucose levels. With this, it is possible to deeply characterize a diabetic person, offering a better understanding of his or her illness evolution, and, going further, develop new strategies to manage DM1. This new and global monitoring makes it possible to extend the “on-board” concept to other features. This well-known approach to the processing of variable “insulin” describes some inertias and aggregated/remaining effects. In this work, such analysis is carried out along with a thorough study of the significant variables to be taken into account/monitored—and how to arrange them—for a deep characterization of diabetic patients. Lastly, we present a case study evaluating the experience of the continuous and comprehensive monitoring of a diabetic patient, concluding that the huge potential of this new perspective could provide an acute insight into the patient's status and extract the maximum amount of knowledge, thus improving the DM1 management system in order to be fully functional.

## 1. Introduction

Diabetes is a metabolic disease, characterized by chronic hyperglycemia resulting from the body's inability to produce and/or use insulin. In a nondiabetic human, glucose homeostasis is a closed-loop system which is able to regulate blood glucose (BG) levels. Unfortunately, this regulation is not possible in patients suffering from type 1 diabetes mellitus (DM1), who do not produce any insulin (a strong hormone able to reduce hyperglycemia), and, therefore, they must exogenously inject this hormone (or wear an insulin pump) in order to reduce their BG levels. Hence, diabetic people must check their BG levels manually (typically through glucose meters which analyze patients' capillary blood samples obtained via finger pricking) several times a day and, based on these data, as well as other factors like meals and exercise, decide when and how much insulin is required to avoid hyperglycemia.

However, a great many new technological advances achieved in recent years offer a new perspective for diabetes management and can constitute a real revolution within this field. An artificial pancreas (AP) is presumed to comprise a continuous glucose monitoring (CGM) device, which is aimed at checking the patient's glucose levels in real time, and a pump which should inject into his or her body the amount of insulin (and glucagon, if applicable) stipulated by a control system based on a closed loop [1-2]. Therefore, CGM has become a cornerstone in diabetes care and future AP prototypes [3], since it can provide the magnitude, tendency, frequency, and duration of glucose level fluctuations almost every minute.

However, some situations cannot be explained solely by the provided CGM data. Sometimes, medical staff may need to question patients about their daily routine or about other specific circumstances for some particular moment, so that they can have a comprehensive understanding. Unfortunately, people remember the context in a subjective way, so doctors and patients cannot analyze the background properly.

Fortunately, beyond CGM, other devices in the field of biometrics allow maintaining a 24-hour monitoring of the patient, recording important information about the person's health, and could also be of relevance in an AP context. In this way, variables like temperature, exercise, heart rate, and perspiration (among others) can be monitored continuously. Advances in biomedical science allow easy and mobile data collection [4-5].

As there is no global monitoring of a diabetic patient, going beyond BG levels alone, there is a necessity to describe the physiological aspects of patients' evolution and current conditions more exhaustively and in much more detail than before. In the present paper, a thorough analysis of the significant variables to be taken into account and monitored in diabetic patients for a fully functional and meaningful DM1 management system will be presented.

In addition, the concept of being “on-board” was widely treated in diabetes research related to the insulin effect. With this, the remaining effect of fast insulin of previous bolus can be taken into account since it should be noted that it can be acting with low intensity for several hours. This idea has been recurrently studied in literature and introduced in a practical way in bolus calculators [6], also taking into account other variables such as the carbohydrate to insulin ratio [7] or the influence of circadian variability in this insulin on-board (IOB) [8]. This interpretation will be extended and justified—as a novelty—to other continuously measured features indicated in this paper.

This paper is organized as follows: Section 2 presents CGM as a cornerstone, with a brief review of the currently offered possibilities. Some other necessary devices for a complete DM1 management system are described in Section 3. Then, as a result of the different possibilities of monitoring, an almost complete characterization of a diabetic patient (the significant variables to be taken into account) is proposed in Section 4.  Section 5 presents a case study and then Section 6 analyzes some challenges to be faced by this idea. The conclusions and possibilities offered by this novel global outlook are given in Section 7.

## 2. Continuous Glucose Monitoring (CGM)

Currently, it is not possible to think of an approach to an artificial pancreas without a CGM device. This mechanism constitutes a revolution in diabetes care, since it can provide for the magnitude, tendency, frequency, and duration of the fluctuations of the glucose levels in diabetic patients [9]. Compared with conventional glucose monitoring (fingerstick, capillary blood glucose monitoring), which provides between three to ten measurements of glucose level per day, CGM can deliver up to one measurement per minute (i.e., 1440 data points per day). Therefore, this sampling frequency is sufficient to be the input of a control system, although Kovatchev et al. indicate [10] that CGM still suffers from some limitations, such as random noise and a transient loss of sensitivity. So, in order to get a complete monitoring of a diabetic person, the first decision is to choose a proper CGM.

A frequent complaint is the time lag between the data provided by the CGM and the real glucose level in the bloodstream of the patient. This is due to the fact that the CGM is not measured directly from the blood: the value that the CGM device offers is an estimate based on the interstitial fluid glucose which exists under the skin, whose level is delayed some minutes with respect to the actual value present in the bloodstream. This fact can be perceived as being a disadvantage. However, recent research has found that this time lag is not more than 5 to 10 minutes [11]. Moreover, mathematical methods can suitably compensate for the lack of accuracy due to this delay. In fact, some tests deployed by Basu et al. confirmed a delay of just 6 minutes [12].

The accuracy of these devices can be evaluated by means of two standards: the mean absolute relative difference (MARD) and the Clarke error grid [13]. The MARD is defined as the result of a mathematical calculation that measures the average disparity between the CGM sensor and the reference measurement. The lower the MARD, the smaller the average error and the more accurate the device can be considered to be. The most accurate CGM devices reach maximum values of the MARD of between 6% and 8%. The main commercialized CGM devices are presented in Table 1.

Dexcom is probably the most accurate CGM system available. In its most recent version (Dexcom G5), a MARD of 9% is reached thanks to the new 505 software, which features the same advanced algorithm as that used in artificial pancreas research. The life of the sensor is seven days, according to the manufacturer's specifications, but users can usually restart it when this expires. The G5 model has several features, such as the possibility of displaying the glucose levels on a smartphone, without the necessity of carrying the Dexcom receiver. The previous version (Dexcom G4 PLATINUM) cannot transmit to a smartphone (just to a Dexcom receiver), but both are able to be connected to an Animas Vibe insulin pump. Other combinations can also be found, like the integration of a t:slim insulin pump with the Dexcom G4 and G5.

Medtronic also offers CGM sensors: Medtronic Enlite, together with their insulin pumps, although it is also possible to use the sensor separately. Its MARD (13.60%) is a little bit higher than that of the Dexcom devices, and the approved life of the sensor is 6 days, although it can be reactivated for up to twelve days. By adding a specific Bluetooth device, it is possible to show the results on a smartphone.

At this moment, the US Food and Drug Administration (FDA) has approved a device with a CGM gadget and an insulin pump, capable of stopping the insulin infusion in case of hypoglycemia (Medtronic 530G) and, recently, the FDA has also approved the Medtronic 670G, which is also able to minimize hyperglycemia thanks to its connection to the CGM gadget.

Abbott now has two products available. The first one is the FreeStyle Navigator II, which was the most accurate CGM device until the new Dexcom algorithm 505 appeared. It presents a MARD of 11%, but, unfortunately, it is only available in certain countries, such as the United Kingdom, the Netherlands, and Israel. It provides a new glucose value every minute, in contrast to the Dexcom G5, where it is necessary to wait 5 minutes for a new value to be offered. The official life of the sensor is 5 days but it may last longer. One disadvantage is that an initial calibration by finger prick glucose monitoring is necessary and, from time to time, a new calibration is required. This is also true for the Dexcom and Medtronic devices.

Abbott has also offered the FreeStyle Libre from the end of 2014 [14]. Strictly speaking, it is not a real CGM device since the glucose information is not transmitted continuously (although it is stored and can be read by scanning the sensor with the receiver), so it is not possible to set alarms for low or high values of glucose. The sensor is placed on the arm and the information can then be transmitted via NFC, known as the “Flash System.” It achieves a MARD of 11.4% and the sensors have a longer life (14 days), but they cannot be restarted. One of its perks is that it does not need to be calibrated.

All of the CGM devices use different algorithms to minimize the delay between the glucose levels present in the bloodstream and those measured under the skin (interstitial fluid) when trying to predict the next value. This delay, mainly when glucose levels are changing fast, results in inaccuracy, the predicted values not being reliable under those circumstances. To avoid this, many improvements have been implemented in CGM. Denoising, enhancement of raw data accuracy, and minimizing error due to the delay introducing a correction are the three main key challenges [15].

The denoising algorithm could be based on an adaptive self-tunable Bayesian smoother [16] able to automatically estimate in real time the signal-to-noise ratio present on the CGM. The other denoising algorithms that can be used here are, e.g., those presented by Palerm and Bequette [17] and Mahmoudi et al. [18].

The enhancement module is proposed in [19] grounded in a stochastic deconvolution-based recalibration algorithm, which rescales the CGM data using a simple linear regressor whose parameters are recalculated every time a new SMBG value is available. The other enhancement/recalibration algorithms that can be exploited in this step are, e.g., those presented by Barcelo-Rico et al. [20] (adaptive calibration) and Kirchsteiger et al. [21] (LMI-based approach).

In the prediction stage, a simple but effective predictor based on an autoregressive model of order one can be used [22], whose key feature is the real-time estimation of the predictor's parameters using a recursive least squares implementation, exploiting a forgetting factor to smartly weigh previously acquired data. Other sophisticated prediction algorithms can be used, including other signals like IOB or physical activity: Zecchin et al. [23] (neural network), Zarkogianni et al. [24] (taking into account physical activity), and Georga et al. [25] (regression models).

## 3. Additional Commercial Smart Devices to Be Included in a Full DM1 Monitoring System

Some new technological advances have brought innovative developments in this area. In the commercial market, it is possible to find new devices which have revolutionized monitoring possibilities.

At the moment, smartphones present a level of adaptability that is unachievable by any other device. They allow us to

1. keep the software responsible to model the dynamics of the system, make a glucose prediction, optimize a solution, and control the process
2. gather information between CGM devices and an insulin pump. The possibilities of connectivity are enormous. Today, a mobile phone is able to communicate not only via 4G but also through Bluetooth, Wi-Fi, NFC, Ant+, etc., which offers a plethora of different choices
3. send data and information to the Cloud, to be either stored or computed (Cloud computing)
4. forward emergency calls in case the patient is at risk
5. update its software when required

Furthermore, smartphones provide a wide range of possibilities in the field of monitoring. Nowadays, the inclusion of accelerometer sensors, gyroscopes, and pedometers allows physical activity to be quantified. Other uses could be to measure physiological features such as heart rate, using camera flash (admittedly with a large uncertainty), and some are able to register the environmental temperature. In any case, these attributes are often used recreationally. In the following paragraphs, some other more specific and more accurate sensors will be described.

These possibilities have been tested in experiments performed by Place et al. [33]; in real time, patients' evolution has been followed from different locations. Other publications have studied the use of a smartphone as a device capable of sending an emergency call, providing the location via GPS in case the patient is at risk [34].

However, there are some doubts as to whether smartphones will be approved as medical devices (class III, high risk). The controller application installed in the phone has to be reliable, so as to avoid conflicts with other applications running on the device. Other circumstances, such as a discharge of the battery or lost connectivity, would have to be prevented. These pros and cons are exposited in Rigla [35].

In another vein, groundbreaking advances in electronics have introduced miniaturization and more powerful innovations in biometrics, the field in which measurable biological characteristics are studied. It is now possible to measure certain variables, most of them being vital signs (such as the heart rate and exercise), in a continuous mode. All of these measurements play a part in the complex balance of blood glucose and must be taken into account. Some years ago, it was difficult to obtain a full-day's data, but, nowadays, devices such as smartbands, smartwatches, and other fitness and medical wearables make this objective easily attainable, offering a lot of useful information [36]. These innovations have potential in the health field due to their ability to collect different types of data and presenting them in a user-friendly way. In addition, they have potential due to their connectivity; they are usually provided with a Bluetooth connection. However, they are restricted by their size, length of battery life, and the fact that these devices are sometimes conceived for non-health care, professional uses. These factors cast some doubts on their accuracy [37], but, in any case, they can provide data of an appropriate order of magnitude. A compilation of the most popular and affordable fitness smartbands is presented in Table 2, along with a summary of their main features. As we can see, these devices can provide a wide range of physiological features and, therefore, can provide a good characterization of the patient. To complete the monitoring, some other medical devices could be worn by the patient in order to measure temperature, blood pressure, etc.

Therefore, although the use of novel devices (accelerometers, electrocardiograms, thermistors, etc.) has been tested, they have only been studied in isolated trials and not included in DM1 management systems. Some of them (but not all) have been used together with CGM devices, sometimes with the sole aim of studying the relationship between BG and other features and sometimes to predict glycemia levels. It should be said that a 24-hour simultaneous monitoring of all the features has not been carried out nor has a study of the types of variables, the correlations between them, ranks of influence on the BG, and a valuable set of features used for modeling the evolution of glycemia.

## 4. Significant Variables to Be Monitored for Complete Diabetes Management

### 4.1. Types of Variables: The Idea of *Something* On-Board

According to the nature of the feature under study and how it changes through time, it is possible to distinguish three different types of variables, each one requiring specific handling.

#### 4.1.1. Pulse Variables

These features interact with the system at one point and then stop. However, this impulse, with a given strength, has the capability to unbalance the system, creating a disruption. In this sense, insulin boluses and meals, and just indicating the exact time when they take place, are pulse variables, and it is known that these features are zero most of the time [38]. They are considered to be discrete events, regardless of the time that it takes to end their influence.

#### 4.1.2. Trending Variables

In this case, it is not only necessary to know the value in the current moment, but, furthermore, a corresponding trend is critical in order to know previous states and then extrapolate (or predict) future values. Examples of this are glycemia, exercise, and heart rate. However, in the scientific literature, it is not possible to find a discussion about how many past values are necessary to take into account in these kinds of variables.

#### 4.1.3. “On-Board” Variables

This is a well-known concept that has been introduced in several previously published works, in order to explain the remaining influence of previous insulin doses (IOB). In this case, the maximum effect has already ceased but the remaining tails of the insulin curve still have a remarkable influence, especially when there were several punctual injections throughout the experiment. In this way, an insulin dose presents its maximum effect at 90 minutes but the mentioned tails could have an influence, even throughout the 6 hours after injection. As these action times can oscillate because of external causes such as exercise, this remaining effect will be the subject of discussion in the next sections. The idea of IOB has been introduced in several previous works, as in [39], where the possibility of a prediction of glucose levels in a 20-minute horizon using proportional integral derivative (PID) controllers is studied. The integral component of the algorithm could cause hypoglycemia, and this is, in essence, the IOB contribution. This paper also presents the concept of “exercise on-board.” After physical activity, the glucose demand from the muscles is still particularly high, in order for them to recover after the effort. This has a permanent and sustained effect in both glycemic demand and insulin sensibility, as previously explained. Therefore, this idea will be modeled with an accumulated amount of exercise in the previous hours. Following the same philosophy, meals not only disturb the system equilibrium in a single moment (ingestion) but they also have a critical influence in a range of several hours. So, meal furthers more of the disruption in glycemia created at the time of eating (which should be better understood as an “announcement”); later, after digestion, the glucose resulting from the metabolic processes is dumped into the blood or, among other destinations, stored as glycogen in muscles and the liver. This glycogen can be directly correlated with the amount of food (mainly carbohydrates) ingested in the hours preceding the measurement, and it can affect the amount of glycemia dumped as glucose into the bloodstream. Hence, the innovative concept of “meal on-board,” as accumulated food, makes sense in explaining this circumstance. The amount of hours needed to consider this fact will need to be discussed deeply. On the other hand, rest level is also an influential variable in glycemia control. A low amount of sleeping time tends to result in rising blood glucose levels because of a higher necessity for stress hormones, which have a hyperglycemic effect. Therefore, it seems reasonable to take account of lack of sleep (sleep deprivation on-board) as well. Even with this idea, it is possible to discuss the influence of the daily routine. Some kind of “schedule on-board” could make sense, and this concept will be developed later. The limit of this sum will be also the object of discussion in future works.

Taking these ideas into account, we can list and describe a complete set of features that can be monitored in order to produce a global description of the patient, capturing his or her physiological history as well as their present condition.

### 4.2. Identified Variables

At present, most of the previous literature on diabetes management systems has only taken into account glycemia and insulin levels, and sometimes an estimation of meals, but it seems reasonable to incorporate additional variables that could also influence glucose levels as far as it is possible to measure or estimate them. It seems that there is a general agreement in using glycemia, insulin, and meal as remarkable variables [40, 41], and the studies with this set of variables are a majority, but it is also possible to find works involving just previous glycemia data as the only variable in use (like those that can be found in autoregressive model approaches [22]) or in [42], just adding insulin to glycemia and using an autoregressive with exogenous term model. There is also a lot of studies in the last years taking into account other variables, mainly exercise, both in silico and in vivo [43], and also considering the possibility of heart rate, temperature, etc. These ideas will be presented in the following lines. In any case, to the best of the authors' knowledge, there is no previous work using a comprehensive characterization of the diabetic patient, examining a global and integral overview.

In this section, a comprehensive list of the significant variables for a complete DM1 control system is analyzed. Some of them have been previously discussed in the scientific literature, but others are presented in this paper for the first time.

#### 4.2.1. Current and Previous Glucose Bloodstream Levels

This is one of the principal variables. In addition to traditional capillary blood monitoring, which consists of discrete glucose values, new CGM devices offer enough accuracy and a good sample frequency [44] to be one of the inputs to a continuous control algorithm.

#### 4.2.2. Insulin

This hormone has an exogenous origin in a diabetic patient, who has to make a decision about the dose to be injected. As it is the variable which primarily governs how much glucose level will decrease due to hypoglycemic response, this could be the main variable requiring optimization. It is necessary to differentiate three ways of taking insulin into account.

*Instantaneous Insulin Input*. It refers to recent doses, especially fast-acting insulin (boluses). This type of insulin usually has an effect for two and a half hours, with a maximum peak effect at 90 minutes.

*Basal Insulin.* It covers 24 hours, compensating for the normal and slow dump of glucose into the blood (due to the action of glucagon) from the glycogen stored mainly within the liver.

*Accumulated Insulin (IOB)*. This concept has been taken into account in some of the scientific literature and means the insulin amount that is currently in the body (and which is, therefore, active). It involves basal insulin and remaining fast insulin, which can be acting with low intensity (but still noticeable) for several hours. It is assumed to have a remarkable effect for the previous eight hours [45], although another research points to an active range of the previous five to eight hours [46].

#### 4.2.3. Exercise

Physical activity increases the muscles' demand for glucose; it is necessary to a normal workout. It also raises blood circulation, so insulin is used up faster and its effectiveness is increased because exercise temporarily makes the cellular walls more permeable, which lets glucose enter the cells more effectively [47]. This could lead to the risk of hypoglycemia and a lower requirement of insulin (however, it should be noted that exercise could cause hyperglycemia in the absence of insulin). Exercise also leads to a reduction of our reservoir of glycogen, which is slowly dumped into the bloodstream throughout the day and relatively faster when hypoglycemia is taking place. Thus, it is possible to make a distinction between three aspects regarding exercise.

*Recent Exercise.* Physical effort has an immediate consequence in glycemia. Thus, it would be reasonable to continuously measure this variable.

*Intensity of Exercise.* The duration can define not only the type of exercise but also the intensity since, for example, the more power or strength are involved, the more there is influence on the insulin's action.

*Accumulated Exercise (Exercise On-Board (EOB)).* Although the increased effect of exercise on both blood glucose and insulin requirements starts to decrease at the same time the physical activity is being performed, a remaining effect can act for up to 48 hours afterwards [48-49]. Moreover, it is proven that regular activity increases the sugar bloodstream equilibrium and reduces the need for insulin [50]. Therefore, the concept of EOB appears to be very necessary if a rigorous glucose prediction method is to be obtained. For this reason, this variable is one of the innovative proposals of this paper.

#### 4.2.4. Meals

What we eat and when we do it have a great influence on blood glucose levels. Food is mainly transformed and absorbed as glucose, which is dumped into the blood, raising glycemia almost instantly. This interaction has been described and modeled by using processing software [51]. Three aspects should be noted regarding meals.

*Notification of Ingestion*. As meals have a fast impact on blood glucose levels, an agile response in an artificial pancreas would be necessary. Unfortunately, the system cannot act as quickly as desired, so any preparation regarding the glucose level increase derived from food intake would be welcome.

*Meal (Carbohydrate Counting)*. How fast and how much the blood glucose level increases depend mainly on the amount of carbohydrates that is present, and so, such information should be registered. It is generally difficult and inefficient to measure the exact amounts that are consumed (patients are sometimes confused when evaluating this parameter because it is a subjective assessment), but, usually, diabetic people have been trained in how to measure meals and carbohydrate counting, based on standardized tables. The speed with which some meals are absorbed and reflected in glycemia has been studied by Bell et al. [52]. In that study, the absorption of fats is estimated at five hours (due to CGM); on the other hand, protein generates a peak at three hours but is less marked and carbohydrate levels peak in about two hours. Of course, meals are a mix of these three components, and the time of the highest total contribution will be very diverse.

*Accumulated Intakes (Meal On-Board (MOB))*. In the long term, food intake increases glycogen reservoirs, and so, on the one hand, the eventual hypoglycemic reaction will be fast, but, on the other, glucose levels will normally be higher due to the slow and continuous dumping of glucagon and consequent liberation of glucose (mainly from the glycogen stored within the liver). Moreover, it should be noted that there can be interactions between the main types of macronutrients when it comes to their absorption, since one can be delayed by the effect of another. For example, the ingestion of fat can slow down the absorption of carbohydrates [53]. In fact, an excessive ingestion of fat can lower the sensitivity of the body to insulin [54]), but, on the other hand, the intake of fiber can slow down the absorption of carbohydrates [55]. Regarding the above statement, the idea of a “meal on-board” concept has arisen and seems reasonable. Unfortunately, to the best of the authors' knowledge, there is no research which takes this idea into account. In this sense, this variable is first presented in the present paper.

#### 4.2.5. Stress

Stress hormones also have a hyperglycemic influence [56]. Adrenaline and cortisol are secreted for several reasons. In this case, two important aspects should be noted.

*Sleep Quality at Night.* Poor quality of rest at night or too few hours of sleep can lead to an altered glucose metabolism and to an insulin resistance (i.e., hyperglycemic effects) [57]. Therefore, another novel variable is proposed: sleep deprivation on-board (SDOB), since the hyperglycemic response derived from sleep deprivation can last for several hours. Thus, an estimation of the number of hours slept should be considered.

*Heart Rate.* An increase of this parameter can be due to several causes. Evidently, this value increases when exercising. However, if such an increase is not related to physical activity, it could also be generated by stressful situations [58]. Moreover, the heart rate can also be indicative of a hypoglycemia episode [59]; the relationship with hyperglycemia has also been studied [60].

#### 4.2.6. Temperature

This parameter can be a symptom of hypoglycemia, becoming lower under these circumstances [61]. However, long-term patients with frequent drops of glucose levels often present a lack of symptoms, not realizing their low-glucose state until the situation is risky. On the other hand, hyperthermia (which is normally the signal of a concurrent illness) usually has hyperglycemic effects [62]. Completing this view, correlation between a hypothermic state could lead to subsequent hyperglycemia [63].

#### 4.2.7. Perspiration

This is another expression of hypoglycemia. As with temperature, this symptom can go unnoticed in long-term patients [64].

#### 4.2.8. Blood Pressure

Arterial hypertension is commonly associated with type 2 diabetes because of the so-called *metabolic syndrome* (which involves insulin resistance) and also with type 1 long-term diabetes patients, generally due to kidney malfunction. Therefore, high blood pressure could point to a poor control of glycemia [65] and lead to complications involving the heart, eyes, kidneys, and blood vessels.

#### 4.2.9. Schedule (Time)

As we are always involved in a set of customs and habits, patients' schedules tend to be similar from one day to the next. It is usually possible to identify a weekly pattern, especially in the case of diabetic patients, since this is generally helpful in controlling their diabetes. Several tasks are usually carried out routinely at the same hour, such as working, eating, practicing sports, or injecting insulin. Thus, by identifying both the hour and the day of the week, it should be possible to anticipate and predict the evolution of the system. Another advantage of recording the schedule is the identification of the basal insulin evolution. New types of slow-acting insulin [66] have an almost flat profile, but it should be noted that it is not 100% flat. Therefore, the hourly absorption and its alterations should be taken into account. In fact, going further, another innovative variable can be considered: schedule on-board (ScOB), which can be seen as the persistence in time of a daily routine, in terms of its impact on glucose levels, when a routine is changed or modified.

#### 4.2.10. Others

Other features that could be considered in the system, in order to reach personalized solutions, could be

- age
- sex
- height
- weight
- body-mass index

There are also some other patient's features, situations, and concurrent (chronic or temporary) illnesses that could affect glucose levels. This issue results in a wide discussion and varies from a patient to another. For instance, special attention has also been developed in some particular diabetic women's circumstances, like pregnancy [67], where the deployment of a specific planning has resulted in an improvement in gestation and childbirth. Continuing with special situations in the life of diabetic women, it is necessary to pay attention to menstruation, menopause [68], or the progression of osteoporosis [69].

In another vein, influence of psychiatric illness has been discussed in [70], concluding the importance of taking into account this coexisting disease. Other concurrent illnesses, especially those derived from diabetes, are also mandatory to bear in mind and be considered as a “variable” to characterize a diabetes patient. In this area, it is possible to name cardiovascular and renal impairment [71] and others like diabetic foot and retinopathy [72], where the importance of considering these simultaneous circumstances and implementing a combined approach has been successfully probed. Although the casuistry of these concurrent situations is so extensive and needs to be studied case by case by specialized medical staff, a deep characterization like that exposed in the present work could provide more exact information and tune exactly a personal diabetes care.

So, although it would be reasonable to add other variables if they have any significance, only the ones that can easily be measured with noninvasive devices are listed above.

**Figure 1:**
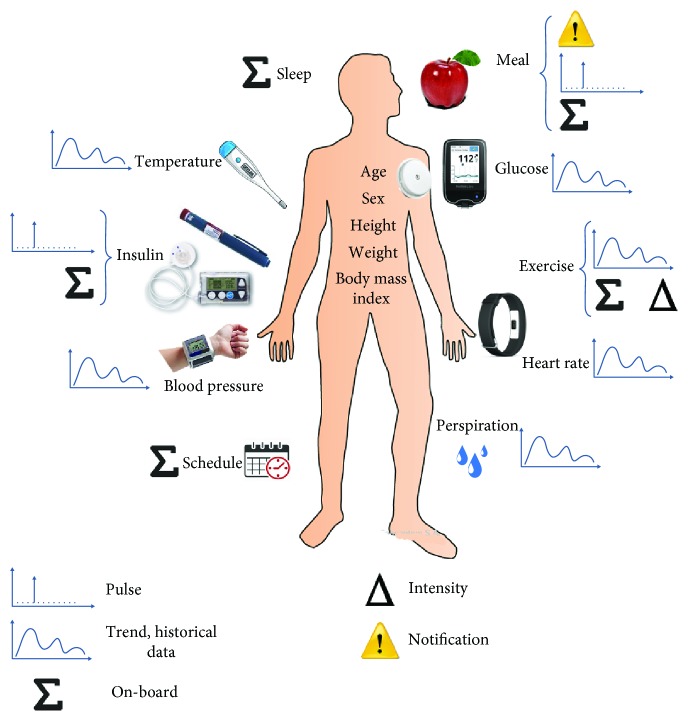
How to characterize a diabetic person using continuous monitoring devices.

Figure 1 summarizes all of the points analyzed in this section.

## 5. Case Study: Results of a Comprehensive Monitoring System

In order to explore the possibilities of the monitoring described above, a case study was carried out. For that, the following devices were used:

1. *Abbott Freestyle Libre*: this groundbreaking device allows accurate and affordable 24-hour glucose monitoring. It also makes it possible to register meals as carbohydrate portions (CP) (one CP is equal to 10 g of carbohydrate) and the insulin dosage (units)
2. *Fitbit Charge HR*: this is an advanced tracking wristband that gives an automatic, continuous heart rate and activity tracking during workouts and throughout the day. It records number of steps, distance, floors climbed, and sleep time. It stays connected wirelessly and can be synchronized with a smartphone and computer to monitor trends. With this smartband the following features were recorded: steps, heart rate, and sleep.

Patients also recorded their meal intake and schedule, indicating periods of work time or playing sports.

Although we listed other features (temperature, blood pressure, and perspiration), they were not monitored due to limitations in the chosen devices, and the additional devices would not have been able to be acquired easily and/or were not affordable. As this was a preliminary study, we considered the measured features with the available tools to be relevant enough to make a first evaluation of the monitoring experience.

The patient studied was a healthy man, 25 years old, DM1 for 12 years, and with diabetes under control. We considered this individual to be a good example for a preliminary study. He was under continuous monitoring for 14 days. In this period, he continued with his daily routine, including work, sports, dietary habits, and insulin dosages. The monitoring was carried out in a passive way, so there was no intervention in his diabetes treatment. A physician checked his medical condition daily, with the aim of assuring the safety of the process.

Data were collected every 5 minutes, although it could have been recorded with a higher frequency. At this rate, the datasheet comprises more than 24,000 measurements; the level of detail achieved is really high. However, in terms of data storage, it is easily affordable.

**Figure 2:**
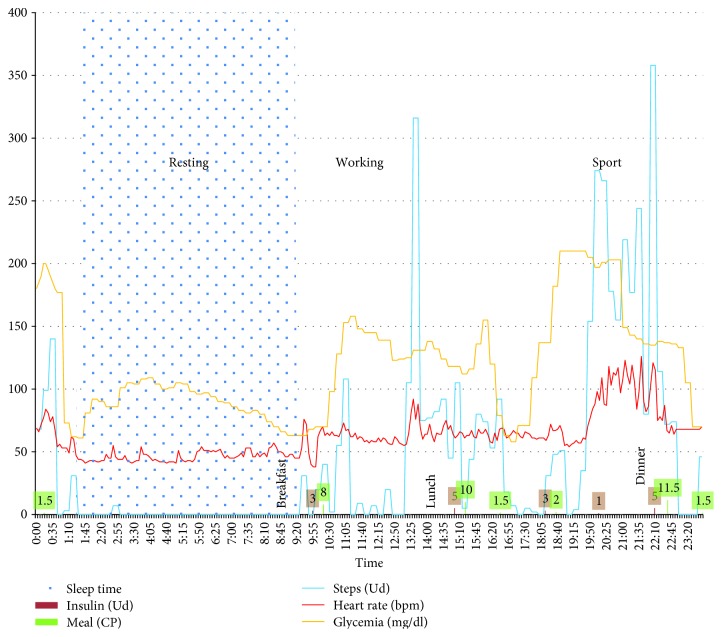
A complete 24-hour monitoring of a diabetic person.

Figure 2 shows the results from a few days and represents an example of the results that can be achieved.

As can be observed, the data provides more information than a simple glucose graph, and, therefore, caregivers can better understand the phenomena that influence the patient's status. With just a glance, it is now possible to understand glycemia evolution just by looking at the relationship with physical activity, for example.

Furthermore, all of the data can easily be transmitted to a smartphone or to the Cloud because of the connections of the CGM and smartbands, or other medical devices, mainly via Bluetooth. With all of these values, the possibilities that arise using machine learning techniques are boundless. It is in this context that the derived “on-board” variables are especially meaningful and have sense to be generated, that this idea encloses aggregative significance, and it is in this situation where the power of this perspective can be completely exploited.

## 6. Challenges to Be Overcome

However, beyond the promising view given above, some challenges still need to be solved. If we want wearable devices to play a crucial role in diabetes monitoring and management, one aspect that has to be ensured is energy supply. Batteries are now able to cover several hours (a few days at best), but we cannot rely on devices which lose power in a short period of time. On the other hand, another challenge is to get the prices of these devices lower, since today some of these devices are unaffordable for some parts of the population. Of course, another issue is to ensure their security and privacy [73]. Finally, it is necessary to deal with the acceptance level of telemedicine. A continuous monitoring that records personal data and provides a complete characterization of the patient could be considered to be interference in their private life. This could lead to an attitude of rejection from the user. These issues are compiled in [74], where it is explained that variables such as age, gender, and previous experiences with telemedicine can modulate its level of acceptance.

As can be seen, some areas need to be improved on, but, on the other hand, new horizons now seem to be opening up.

Modeling techniques based on artificial intelligence methods open up the possibility of predicting glycemia and anticipating actuation. This will help us follow the different stages of the so-called “Kowalski Path” [75] which needs to be followed in order to obtain an artificial pancreas. Unfortunately, such methods have not been studied taking into account the preceding considerations. In this sense, the fields of work that have been opened up are immense and promising. Implementing a predictive model in order to forecast the evolution of the patient is something highly desirable. It could anticipate risky situations or even provide decision support. Unfortunately, the development and validation of models using machine learning techniques are far from complete, laying aside in all the studies some features that could improve the model.

A complete monitoring leads to an enormous amount of data that needs to be transformed into knowledge. In this regard, it is necessary to study complete technological platforms that allow proper diabetes management, taking advantage of improvements in information and communication technologies (ICT); however, this is outside the scope of this paper. These architectures make no sense if they are partial and consider only some features of the patient's status. In this sense, this paper is a good beginning to lay down the basis for such a purpose, in order for future research to develop a complete ICT framework for efficient DM1 management.

Developing a fully operational ICT-based platform could help in information exchange between caregivers and medical staff, provide a better way to manage emergency situations, allow pattern identification, and optimize solutions to deal with DM1.

In addition to these advantages, the clearer and more complete explanation of the patient's evolution is a good benefit, as far as it results in a physician's better understanding of the context of their illness and thus improves the chosen treatment.

This idea of using ICT and developing a telemedicine framework has been studied and put into practice many times, for instance, as early as 2008, the INCA (Intelligent Control Assistant for Diabetes) system [76] or the DIAdvisor [77] in 2010, with excellent results. The possibilities of a remote blood glucose monitoring have also been exposed more recently in [78]. All of these issues have been specifically analyzed in a previous work of the authors [79], with an extended evaluation of the historical development of theses ICT-based platforms, their pros and cons, and a proposal of a complete diabetes telemedicine system in an ICT environment.

## 7. Conclusions

Our world is changing in a very remarkable way and continuously being updated due to advances in technology. All of these improvements have become revolutionary in many fields, making life easier in a lot of respects. In the struggle to obtain a definitive way to monitor and control diabetes, some improvements have occurred due to the development of new devices like those with CGM.

However, this monitoring is not enough to fully define the evolution of a diabetic person. There is a wide set of subjective features that need to be taken into account in the patient's mind. This also implies that caregivers cannot analyze all of the influences on a patient's condition and this can lead to a wrong interpretation.

As we have shown, it is now possible to find wearable monitoring systems that provide a lot of information about the human body. Smartbands, new medical devices, and CGM harvest thousands of measurements per day, which was something unthinkable some years ago. Moreover, as presented in this paper, a wide and definitely complete range of novel variables needs to be monitored for a full diabetes control system, and, furthermore, the idea of “on-board” has been extended to other features. This compilation had not been proposed so completely before and allows the description of patients in a global, comprehensive way. As an example of the possibilities of this monitoring, a case study has been discussed.

In future work, we will take advantage of these new perspectives. The content of this paper could contribute to the development of new information and communication technology (ICT) platforms for a global management system of DM1, as well as the development of a complete and totally operational artificial pancreas.

## Figures and Tables

**Table 1:** Current commercialized CGM sensor systems.

Company	Model	Features		MARD
Abbott	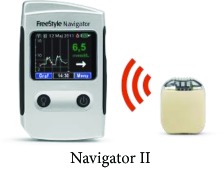	Trend marks Rate-of-change alerts Alarms (hyper/hypo) 5-day lifetime	Calibration recommended up to 72 h	14.5% [26]
	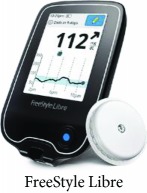	Trend marks No alerts or alarms 14-day lifetime NFC communication with smartphone	Read approaching meter to sensor (NFC technology) No calibration needed	11.4% [27]

Dexcom	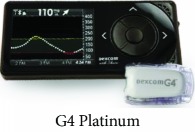	Trend marks Rate-of-change alerts Alarms (hyper/hypo) 7-day lifetime	Remote monitoring Calibration every 12 h	13% [28] (Original algorithm)
	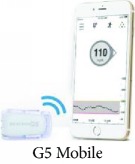	Trend marks Rate-of-change alerts Alarms (hyper/hypo) 7-day lifetime	Remote monitoring Wireless communication with smartphone Calibration every 12 h	9% [29]

Medtronic	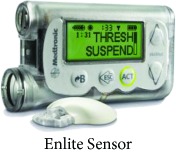	Trend marks Rate-of-change alerts Alarms (hyper/hypo) 6-day lifetime	Integration with Medtronic 530G insulin pumps Calibration every 12 h	13.6% [30]
	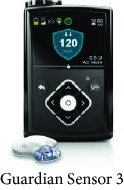	Trend arrows Rate-of-change alerts Alarms (hyper/hypo) 7-day lifetime	Integration with Medtronic 670G insulin pumps Calibration every 12 h	9.1% [31]

Senseonics	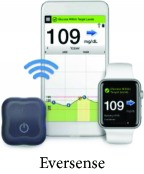	Trend arrows Rate-of-change alerts Alarms (hyper/hypo) 90-day lifetime	Communication with smartphone Sensor inserted under the skin	11.6% [32]

**Table 2:** Main currently commercialized smartbands.

Company	Model	Sleep	Distance	Steps	Calories	Waterproof	Heart rate	Interface	Battery
Fitbit	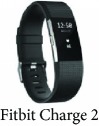	✔ High accuracy	✔	✔ High accuracy	✔	Small	✔	iPhone Android	4–7 days
	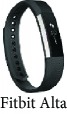	✔	✔	✔	✔	Small	✖	iPhone Android	4–7 days
	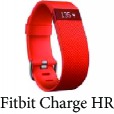	✔	✔	✔	✔	1–2 m	✔	Web iPhone Android	1–2 weeks
	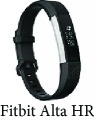	✔	✔	✔	✔	Small	✔	Web Android	1–2 weeks

Polar	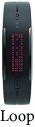	✔	✔	✔	✖	Small	✖	iPhone Android	1–2 weeks

Garmin	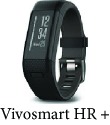	✔ High accuracy	✔	✔ High accuracy	✔	5 m	✔	iPhone Android	4–7 days
	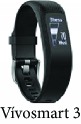	✔	✔	✔	✔	Small	✔	iPhone Android	4–7 days

Jawbone	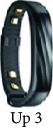	✔ High accuracy	✔	✔ High accuracy	✔	Small	✔	iPhone Android	1–2 weeks

Xiaomi	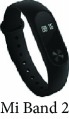	✔ High accuracy	✔	✔ High accuracy	✔	Small	✔	iPhone Android	2–3 weeks
